# Dirty Fish Versus Squeaky Clean Mice: Dissecting Interspecies Differences Between Animal Models of Interferonopathy

**DOI:** 10.3389/fimmu.2020.623650

**Published:** 2021-01-15

**Authors:** Holly A. Rutherford, Paul R. Kasher, Noémie Hamilton

**Affiliations:** ^1^ The Bateson Centre, Institute of Neuroscience, Department of Infection, Immunity and Cardiovascular Disease, The University of Sheffield, Sheffield, United Kingdom; ^2^ Lydia Becker Institute of Immunology and Inflammation, Division of Neuroscience and Experimental Psychology, School of Biological Sciences, Manchester Academic Health Science Centre, The University of Manchester, Manchester, United Kingdom; ^3^ Geoffrey Jefferson Brain Research Centre, Manchester Academic Health Science Centre, Northern Care Alliance & University of Manchester, Manchester, United Kingdom

**Keywords:** type I interferonopathy, Aicardi-Goutières syndrome, autoimmune disease, RNASET2, zebrafish, mouse, white matter disorders, leukodystrophy

## Abstract

Autoimmune and autoinflammatory diseases are rare but often devastating disorders, underpinned by abnormal immune function. While some autoimmune disorders are thought to be triggered by a burden of infection throughout life, others are thought to be genetic in origin. Among these heritable disorders are the type I interferonopathies, including the rare Mendelian childhood-onset encephalitis Aicardi-Goutières syndrome. Patients with Aicardi Goutières syndrome are born with defects in enzymes responsible for nucleic acid metabolism and develop devastating white matter abnormalities resembling congenital cytomegalovirus brain infection. In some cases, common infections preceded the onset of the disease, suggesting immune stimulation as a potential trigger. Thus, the antiviral immune response has been actively studied in an attempt to provide clues on the pathological mechanisms and inform on the development of therapies. Animal models have been fundamental in deciphering biological mechanisms in human health and disease. Multiple rodent and zebrafish models are available to study type I interferonopathies, which have advanced our understanding of the human disease by identifying key pathological pathways and cellular drivers. However, striking differences in phenotype have also emerged between these vertebrate models, with zebrafish models recapitulating key features of the human neuropathology often lacking in rodents. In this review, we compare rodent and zebrafish models, and summarize how they have advanced our understanding of the pathological mechanisms in Aicardi Goutières syndrome and similar disorders. We highlight recent discoveries on the impact of laboratory environments on immune stimulation and how this may inform the differences in pathological severity between mouse and zebrafish models of type I interferonopathies. Understanding how these differences arise will inform the improvement of animal disease modeling to accelerate progress in the development of therapies for these devastating childhood disorders.

## Introduction

Type I interferons (IFNs) play an essential role in the antiviral innate immune response—protecting the host from productive viral infection before the development of adaptive immune response to pathogens (1, 2). Upon detection of foreign nucleotides in the host, canonical type I IFN signaling activates a number of pathways—ultimately leading to upregulation of interferon-stimulated genes (ISGs) and wide-ranging effects comprising host defense (2, 3). However, while type I IFN signaling is protective in response to active viral infection, aberrant activation of this pathway has been suggested to occur in autoinflammatory disease, triggered by genetic mutations in the host (1, 4).

The association between upregulation of type I IFN and autoimmune/autoinflammatory disease was first proposed following the observation of overlapping phenotypes between such disorders and congenital HIV-1 infection (5). Following subsequent genetic characterization, a distinct grouping of disorders has emerged, in which disturbance of the homeostatic control of type I IFN response—and subsequent upregulation of ISGs—due to Mendelian mutations is central to pathogenesis (4, 6, 7). Now collectively referred to as the type I interferonopathies, this group includes the chronic autoimmune disease systemic lupus erythematosus (SLE), the inherited encephalopathy Aicardi-Goutières syndrome (AGS) and a range of often rare but devastating conditions (4).

In this review, we focus specifically on AGS and the closely related RNaseT2-deficient leukodystrophy. Alongside the prominent inflammatory phenotype typical of type I interferonopathies, these disorders present with devastating neurological phenotypes which are not only debilitating to patients but have proven particularly difficult to recapitulate in animal models (8). Accurate, valid animal models are essential for the development of novel therapies: thus far, the translational impact of animal models of interferonopathies has been vastly limited by the lack of neuropathology in preclinical settings. Here, we summarize the human phenotype of AGS and RNaseT2-deficient leukodystrophy and provide a brief overview of the human genetics involved in these disorders. For each of these interferonopathy-linked genes, we analyze the relevance of existing animal models to the human condition, comparing and contrasting models of different species. Finally, we propose that key environmental modulators—namely, early life viral exposure—may account for the differences in phenotype across species and suggest how this theory could be tested to inform our understanding of the human condition.

## The Genetics of Aicardi–Goutières Syndrome and Related Interferonopathies

Of all the conditions now recognized as type I interferonopathies, AGS is perhaps among the most extensively characterized. Although rare, patients with this progressive encephalopathy present with severe intellectual, speech and motor disability in infancy—often mimicking aspects of congenital viral infection (7, 9). Clinical phenotypes become apparent within the first year of life for most patients, with disease onset thought to occur *in utero* in up to one in five patients (10). Although symptoms and severity vary, most individuals with AGS present with one of several “classical” clinical presentations—most commonly including white matter disease, intracranial calcification and microcephaly—although additional genetic subtype-specific pathological hallmarks have also been characterized (**Table 1**) (7). Regardless of mutation, patients with AGS show consistent and significant upregulation of type I IFN and ISG expression—supporting their classification as a type I interferonopathy.

**Table 1:** Summary of animal model phenotypes in interferonopathy research.

	Human	Mouse	Zebrafish
**TREX1 *(AGS 1)* [loss-of-function]**			
*Immunological*	Upregulation of ISG transcripts (10, 11)	Severe multiorgan inflammation; inflammatory myocarditis; IFN-dependent pathology (12–14)	*[n/a]*
*Neurological*	White matter abnormalities and intracranial calcification; abnormal sensorimotor development (7)	None reported (14)	*[n/a]*
**RNASEH2A, -B and -C (AGS2, -3 and -4) [loss-of-function]**			
*Immunological*	Upregulation of ISG transcripts in some patients (10, 11)	Evidence of upregulated ISG expression (15–17)	*[n/a]*
*Neurological*	White matter abnormalities and intracranial calcification; abnormal sensorimotor development; non-syndromic spastic paraparesis (7, 18, 19)	None reported (16)	*[n/a]*
**SAMHD1 (AGS5) [loss-of-function]**			
*Immunological*	Upregulation of ISG transcripts (10, 11)	Upregulation of ISG transcripts, not reflected at protein level (20–22)	Upregulation of type I IFN, ISGs, and other genes involved in innate immunity (23)
*Neurological*	White matter abnormalities and intracranial calcification; abnormal sensorimotor development; intracerebral, large vessel disease (intracerebral hemorrhage and infarcts) (7, 24)	None reported (20–22)	Cerebral hemorrhage; cerebral oedema (23)
**ADAR1 (AGS6) [loss-of-function]**			
*Immunological*	Upregulation of ISG transcripts (10, 11)	Upregulation of ISG transcripts; embryonic lethal (25–30)	Increased expression of ISGs and other genes involved in innate immunity (23)
*Neurological*	White matter abnormalities and intracranial calcification; abnormal sensorimotor development; bilateral striatal necrosis; non-syndromic spastic paraparesis (7, 19, 31)	None reported; embryonic lethal (25–27)	Severe developmental defects (23)
**IFIH1 (AGS7) [gain-of-function]**			
*Immunological*	Upregulation of ISG transcripts (10)	Severe multiorgan inflammation; upregulated ISG expression (32)	No published gain-of-function mutation; loss-of-function mutation restores expression of immune-regulated genes to wild type levels in mutants with upregulated interferon response (33)
*Neurological*	White matter abnormalities and intracranial calcification; abnormal sensorimotor development; non-syndromic spastic paraparesis (7, 19)	None reported (32)	None reported (33)
**RNASET2 [loss-of-function]**			
*Immunological*	Phenotype mimicking cytomegalovirus infection (34) Upregulation of ISGs in some patients (18, 35)	Neuroinflammation [see below]; no evidence of systemic inflammation (36)	Upregulation of ISG transcripts including isg15 (37, 38)
*Neurological*	White matter abnormalities, intracranial calcification; subcortical cysts (34)	Enlarged hippocampus and prefrontal cortex; increased reactive astrocytes in hippocampus (36)	White matter abnormalities beginning during embryogenesis (microglial dysfunction); locomotor defects (37, 38)

**Figure 1:**
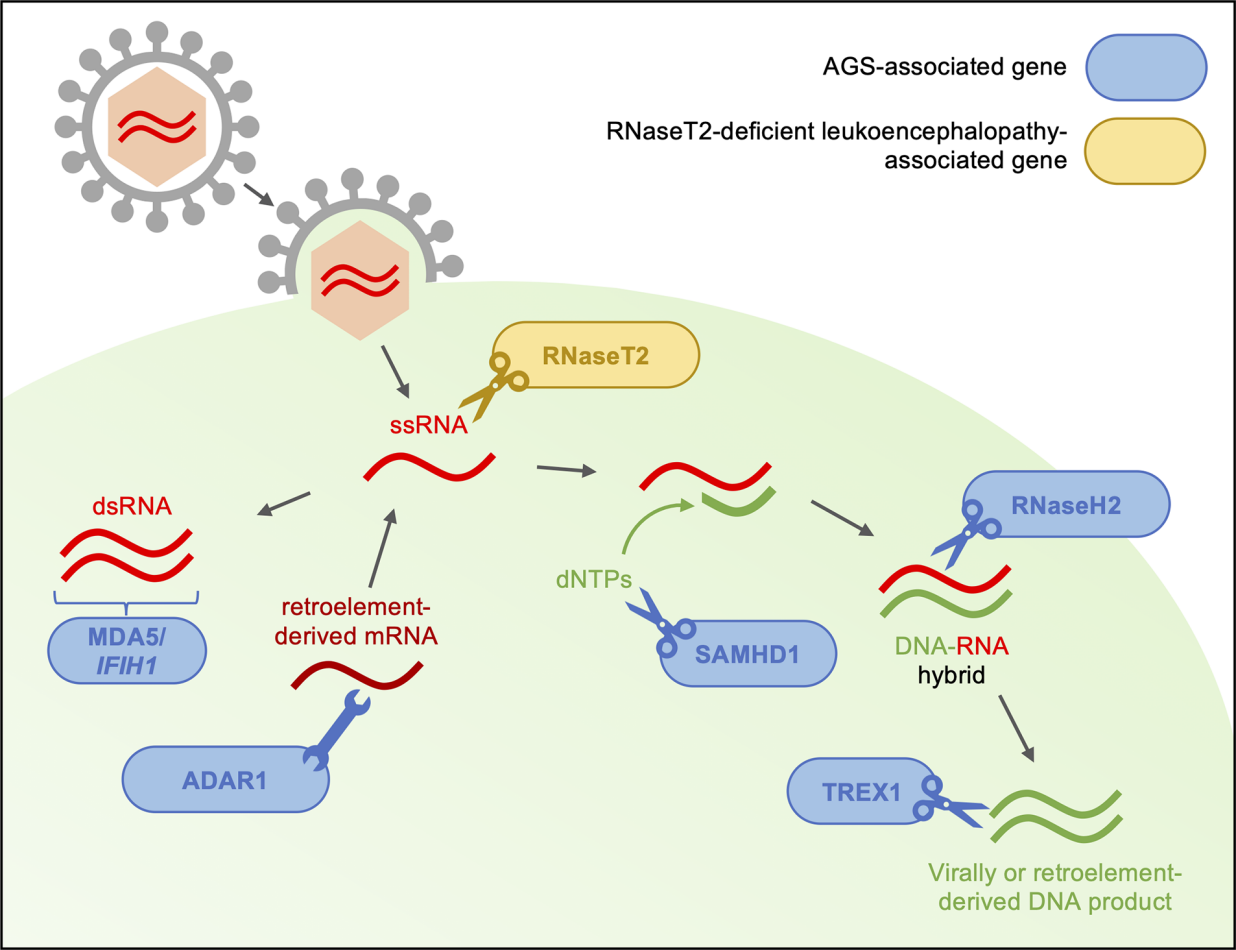
Type I interferonopathy-associated genes are involved in the sensing and metabolism of viral RNA. Genes linked to AGS and RNaseT2-deficient leukoencephalopathy are thought to encode proteins involved in the restriction of reverse transcription of both viral- and endogenous retroelement-derived DNA. The *IFIH1* gene product, MDA5, is involved in the antiviral response through the recognition of dsRNA and subsequent production of type I interferon. With interferon-inducible expression, ADAR1 acts as a suppressor of type I IFN signaling through its RNA editing activity. RNaseT2 is a lysosomal hydrolase involved in RNA metabolism. SAMHD1 limits reverse transcription though degradation of deoxynucleotides necessary for complementary DNA strand formation. Among other roles in DNA synthesis and repair, RNaseH2 is thought to degrade the RNA component of DNA-RNA hybrids formed during reverse transcription. Finally, TREX1 is involved in the regulation of the interferon-stimulatory DNA response after viral infection through metabolism of virally derived nucleotides. In the absence of functioning AGS or RNaseT2 proteins, accumulation of immunostimulatory deoxyribo- and ribonucleotides may trigger upregulation of type I interferon pathway (6, 7, 12, 25, 32, 37, 39–43).

To date, seven genes have been identified as the genetic trigger for different subtypes of AGS (AGS1–7), each of which encode proteins involved in detecting or metabolizing nucleic acids and particularly in restricting reverse transcription (see **Figure 1**) (7). Along with the viral-like phenotype of AGS patients, this has led to the hypothesis that type I IFN is triggered by the accumulation of self-derived nucleotides from endogenous retroelements in some AGS patients (44). In support of this, preclinical and initial clinical studies have suggested that reverse transcriptase inhibitors (RTIs) may have clinical benefits in AGS (7, 45, 46). However, the effects of RTIs on neurological phenotype remains unclear: firstly, because the animal models utilized in these preclinical studies do not develop neuroinflammation even before treatment and, secondly, because the patients enrolled in clinical trials had significant impairments at baseline, such that improvement was not to be expected trials (7, 46). Arguably the core component of disease, much remains to be understood about the neuropathology of AGS: how it develops, why it varies between patients and, ultimately, how it can be treated.

In addition to AGS1–7, mutations in *RNASET2* have been linked to a closely related interferonopathy in human patients, with a similar neurological and inflammatory phenotype: RNaseT2-deficient leukoencephalopathy. Much like in AGS, patients with mutations in *RNASET2* present with psychomotor impairments, micro- or normocephaly and spasticity—mimicking congenital cytomegalovirus infection (34, 35). As with AGS-associated genes, RNaseT2 is involved in the sensing of nucleic acids—either endogenous or virally derived. Thus, we believe discussion of RNaseT2-deficient leukodystrophy alongside AGS in the context of interferonopathy here is warranted.

Recapitulating AGS and RNaseT2-deficient leukoencephalopathy phenotypes in animal models could provide crucial insights into neuropathology and invaluable preclinical therapy development. However, as mentioned above, the translational impact of these models remains minimal—largely as many of these models do not develop neurological abnormalities. Understanding why preclinical models have failed to fully recapitulate the human phenotype is essential to furthering our understanding of interferonopathy progression.

## Animal Models of Interferonopathies

With such distinct and well-documented genetic underpinnings, it is unsurprising that preclinical research in AGS and related interferonopathies has focused on monogenic animal models. Much of this research has been conducted in rodent and zebrafish models of disease—with mouse models largely dominating the field until recent years. The overall merits of these model organisms in interferonopathy and, specifically, leukodystrophy research have been reviewed elsewhere (8). Here, we provide an overview of the phenotypes of currently available mouse and zebrafish models by gene, focusing on their relevance to the human clinical presentation (**Table 1**).

### TREX1 (AGS1)

With roles in antiviral response and metabolism of intracellular RNA, the gene encoding 3’ repair exonuclease, TREX1, was the first to be linked to AGS (12, 39). Accordingly, the phenotype of Trex1-deficient mice is perhaps the most extensively characterized of all AGS-associated models. *Trex1^-/-^* mice develop multiorgan inflammation—predominated by inflammatory myocarditis—and limited survival due to circulatory failure (13, 14). Crucially, however, *Trex1^-/-^* mice do not display any evidence of neuropathology—in fact, the brain appears to be one of the few tissues which does not develop an inflammatory phenotype (14). It is unclear why the brain appears to be protected from pathology in this manner—limiting the utility of Trex1-deficient mice as a preclinical model of AGS.

In addition to their links with AGS, mutations in *TREX1* (and *SAMHD1*, discussed below) have been associated with other autoimmune disorders which are characterized by a more widespread inflammatory phenotype and less prominent neurological involvement (4, 47–50). Although clinical overlap between SLE and AGS has been reported in some patients, it remains unclear why some patients with *TREX1* mutations develop a phenotype dominated by neuroinflammation and others do not (5, 51–53). Therefore, *Trex1^-/-^* mice may better reflect the SLE phenotype and should therefore be considered a more useful model of this disorder, rather than AGS.

Perhaps the development of complementary zebrafish models could further elucidate the role of TREX1 in AGS neuropathology. Human *TREX1* and *TREX2* are co-orthologous with zebrafish genes *trex3* and *trex4*. Interestingly, *trex3* expression is upregulated in zebrafish injected with type I IFN, suggesting this gene is an ISG and may act as a functional orthologue to TREX1 (54). Hence, experimental manipulation of *trex3* expression may be informative about the role of its human equivalent in AGS.

### RNase H2A, -B, and C (AGS 2, 3, 4)

Composed of three subunits, the ribonuclease H enzyme (RNaseH2) complex has roles in DNA synthesis and repair, including LINE-1 retrotransposition (44). Together, mutations in *RNaseH2A, -B* and *-C* account for over 50% of cases of AGS—demonstrating a clear role for this gene in interferon-induced pathogenesis (55). While characterization of an *rnaseh2* zebrafish model is yet to be published, several mouse models have been generated to dissect the role of RNaseH2 in the neurological and inflammatory phenotype of AGS—yet, none have fully recapitulated the human disease (15–17, 56, 57). While hypomorphic models (with point mutations in single subunits) have demonstrated some upregulation of ISG expression, perhaps the model with the greatest face validity is the RNaseH2^ΔGFAP^ mouse—a brain-specific knockout, lacking RNase H2 specifically in astrocytes and neurons (15–17). Astrocytes cultures from these animals demonstrated increased ISG transcript levels, along with signs of DNA damage and premature senescence—consistent with a type I interferon response (16). However, this was not accompanied by any evidence of neuroinflammation or overt neurological phenotype in the whole animal—failing to recapitulate the key components of human disease.

### SAMHD1 (AGS5)

In accordance with the reverse transcription theory of AGS pathogenesis, *SAMHD1* encodes a protein involved in restricting viral DNA synthesis—degrading the intracellular deoxynucleotides needed for reverse strand formation and therefore limiting both viral DNA replication and retrotransposon transcription (7, 58, 59). Mutations in *SAMHD1* are thought to account for around 13% of AGS mutations and have been linked to several other interferonopathies—demonstrating a clear link between SAMHD1 dysfunction and autoimmunity (4, 10, 60).

While there is considerable variation in the clinical phenotypes of AGS patients—regardless of their genotype—patients with *SAMHD1* mutations present with a somewhat distinct phenotype, with intracerebral large vessel disease being a hallmark of pathology which can present as cerebral arterial stenosis, intracerebral hemorrhage or other cerebrovascular abnormalities such as moyamoya presentation (7). Cerebral hemorrhage has been recapitulated by zebrafish models, following knockdown of *samhd1* using antisense morpholinos (23). These animals present with swelling of the hindbrain ventricle and cerebral hemorrhage during embryogenesis. This neurophenotype is accompanied by upregulated expression of a panel of genes known to be involved in IFN-mediated antiviral response—including *isg15* (an interferon-stimulated gene known to be involved in the zebrafish immune response)—suggesting knockdown of *samhd1* induces an interferon response in zebrafish models mimicking the human state (61).

In contrast, SAMHD1 knockout mice fail to develop both the neurological and immunological components of the human AGS neurological phenotype—remaining healthy into adulthood with no evidence of autoinflammatory pathology (20–22). While ISG transcripts are upregulated in these animals, this is not mirrored at a translational level—with no observable difference in ISG products or IFN proteins across multiple tissues, alongside a lack of inflammatory pathology in the heart and skin.

It is curious that reduced (but not abolished) expression of *samhd1* in zebrafish leads to a more extreme neurological phenotype than complete knockout in mouse models. It has been suggested that the function of murine Samhd1 may differ from that of the human and zebrafish orthologue—perhaps with lesser involvement in the innate immune response to nucleic acids in mice than the other species (22). Conversely, it is possible that a compensatory mechanism exists in the mouse that is not present in humans or zebrafish, suppressing the IFN response and preventing the formation of a neurological phenotype as might be expected in knockout mice (23). Nonetheless, the finding that zebrafish models of AGS better recapitulate the human SAMHD1-linked neurological phenotype relative to their murine counterparts raises interesting questions about the use of these species in interferonopathy modeling.

### ADAR1 (AGS6)

Like SAMHD1, ADAR1 has been proposed to be involved in the restriction of reverse transcription due to its intrinsic RNA editing activity (7). Unlike other ADAR isoforms, *ADAR1* expression is interferon-inducible, with a prominent role as a suppressor of type I IFN signaling (6, 25, 40). Both mouse and zebrafish models have been generated to dissect the role of ADAR1 in interferonopathy pathology, with limited success.

Several *Adar1* knockout and mutant lines have been investigated in mice, many of which die during embryogenesis or early life (25–30). Characterization of embryonic lethal *Adar* null mutants revealed upregulation of ISG expression, which could be partially rescued through mutation of *Ifnar1* (IFN-α and -β receptor 1) and fully rescued by mutation of *MAVS*—a key adaptor protein involved in antiviral response—suggesting knockout of *Adar* induces a type I IFN response (25, 28).

A similar immunological phenotype has been reported in zebrafish with impaired expression of the zebrafish orthologue of ADAR1, through the use of antisense morpholinos (23). Although not fully characterized, *adar* ATG and splice morphants display increased expression of a panel of innate immune genes, including the ISGs *isg15*, *irf7*, and *stat1b*.

In contrast to animal models focusing on other AGS-associated genes, it seems that zebrafish and mouse models of ADAR1 dysfunction present with phenotypes that are, in many instances, arguably more severe than the human condition. It is notable that, in mammals, three proteins exist within the ADAR gene family: two of which are thought to have roles in A-to-I editing within the central nervous system (ADAR1 and ADAR2), while the other is thought to have no intrinsic enzymatic activity (26, 27, 62, 63). While each of these expresses discrete functions and ADAR1 is thought to be responsible for most of the editing activity, it has been suggested that ADAR2 may be able to partially compensate in human patients with *ADAR1* mutations—preventing the severe phenotypes and embryonic mortality observed in zebrafish and mouse models (63, 64). Although mice and zebrafish also possess three ADAR genes, it is possible that the distribution of enzymatic activity across these three isoforms differs across species, such that the remaining proteins are less able to compensate for the loss of functioning ADAR1/adar in the models discussed above than in humans (65). Any differences in ADAR function across species in the context of interferonopathies remain speculative at this stage—nonetheless, the disparity between zebrafish, mouse and human phenotypes here highlights an extra layer of complexity when modeling even monogenic disorders.

### IFIH1 (AGS7)

Of all of the AGS-associated genes, mutations in *IFIH1* were most recently identified in AGS patients—with *IFIH1* being the only AGS-associated gene to present with gain-of-function mutations in patients. *IFIH1* encodes the RIG-I-like receptor MDA5, which has a prominent role in antiviral defense through the detection of double stranded RNA and downstream activation of type I interferon response (32, 41). Patients with *IFIH1* mutations develop phenotypes typical of AGS, including severe developmental delays, progressive microcephaly and upregulation of ISG transcription (**Table 1**) (41).

The role of MDA5 in activation of the innate immune response is supported by published work with zebrafish loss-of-function crispants (33). While lack of functioning mda5 alone did not lead to significant changes in innate immunity-associated genes (including *irf7* and *stat1b*), mutation of *mda5* was sufficient to restore expression of these genes to wildtype levels in animals with an already upregulated interferon response (*zbtb24* mutants) (33). The immune phenotype of these *zbtb24* mutants is thought to be triggered by increased levels of double stranded RNA transcripts in the cytoplasm—supporting the role of Mda5 as an essential mediator of the innate immune activation in response to RNA. However, to our knowledge, no zebrafish models of *mda5* gain-of-function—the genotype of greatest relevance to AGS—have been published thus far.

In contrast, MDA5 gain-of-function rodent models have been characterized. In accordance with the autoimmune phenotype of patients, *Ifih1* mutant mice develop severe multiorgan inflammation—including nephritis and calcification of the liver—alongside reduced survival and upregulated expression of IFN and ISG transcripts (32). However, despite such a severe systemic inflammatory response, an overt neuroinflammatory phenotype has not been reported in *Ifih1* rodent models. Thus, until a gain-of-function zebrafish model is generated with a view to recapitulating AGS, much remains to be understood regarding the role of *IFIH1* in interferonopathies, particularly in relation to neuropathology.

### RNASET2

Much like the monogenic mutations linked to AGS, the association of mutations in *RNaseT2* with a similar interferonopathy has led to the generation of animal models exploring the function of this gene. As previously discussed, patients with mutations in *RNaseT2* present with clinical and radiological phenotypes closely mimicking those seen in AGS—suggesting the possibility of shared pathogenesis (35). Indeed, similar to AGS-linked genes, the lysosomal enzyme RNaseT2 is involved in restriction of reverse transcription through the metabolism of virally- or endogenously-derived single-stranded RNA (**Figure 1**) (42).

While no RNaseT2 mouse models have been published, both zebrafish and rat models have variably recapitulated the human phenotype. *RNaseT2* knockout rats develop a robust neuroinflammatory phenotype—with enlarged prefrontal cortex and hippocampus, accompanied by increased numbers of reactive astrocytes in the hippocampus (36). Accordingly, these animals show impaired object recognition, but are otherwise viable—with normal life expectancy and motor function. However, the overall inflammatory phenotype of these animals remains unclear—no evidence of systemic inflammation has been reported in *RNaseT2^-/-^* rats. Crucially, these animals also fail to recapitulate the key hallmark of RNaseT2-deficient leukodystrophy pathology: white matter abnormalities.

White matter lesions, subcortical cysts and calcification are central to the pathogenesis of RNaseT2-deficient leukodystrophy, contributing to the devastating psychomotor impairments observed in the clinic (34). Use of magnetic resonance imaging (MRI) has demonstrated that adult *rnaset2* mutant zebrafish develop robust white matter lesions, with further work suggesting white matter abnormalities begin during zebrafish embryogenesis, as reflected in microglial dysfunction just five days post-fertilization (37, 38). Similar to patients, *rnaset2* mutant zebrafish display locomotor defects from early development into adulthood and significantly reduced survival (38). Beyond the neurological phenotype, *rnaset2* mutants display increased expression of ISG transcripts—including *isg15*—mimicking the viral-like phenotype of patients (37).

Thus, while only three of the genes discussed above have been modelled in zebrafish to date, it would seem that fish models are able to recapitulate neurological phenotypes of type I interferonopathies, while their rodent counterparts are somewhat spared from neuropathology. Of each of the rodent models utilized above, only the *RNaseT2* knockout rat develops evidence of neuroinflammation, and even this appears to be limited to the hippocampus—with overall white matter integrity and sensorimotor function preserved. It is notable that rats possess only a single-copy of *RNaseT2*, while mice possess an additional copy of the RNaseT2-encoding gene—highlighting the importance of assessing the genetic background of the model system before considering its relevance to the human phenotype (36). Nonetheless, the consistent differences between zebrafish and murine models pose interesting challenges for interferonopathy modeling in these species.

## What Can We Learn From Interspecies Differences in Animal Models of Interferonopathy?

Despite the crisis in translation of preclinical research into therapeutic advances, rodent models have remained at the forefront of immunological research for decades (66–68). Mice have long since been considered of sufficient evolutionary similarity to humans to act as a relevant model of research. Yet, in the field of interferonopathy modeling, it seems the zebrafish—a species more evolutionarily distant from humans—arguably better recapitulates clinical phenotypes, with particular relevance to the neurological symptoms at the core of AGS and RNaseT2-deficient leukodystrophy. What, then, is the missing link between zebrafish and mice in interferonopathy research?

### Age of Assessment

One crucial consideration when assessing the face validity of preclinical models—particularly those which model diseases which manifest almost consistently during early life—is the age at which the animals are screened for pathology (10). In patients with AGS, clinical phenotypes frequently emerge during the first year of life, with prenatal disease onset thought to occur in up to one fifth of patients, suggesting analysis of disease phenotypes may be most relevant during early development (10). However, it should be noted that, for the models discussed above, mouse phenotypes were assessed postnatally or in early adulthood, while zebrafish were often screened during embryogenesis or larval stages. This is, in part, due to the intrinsic features of the species used: due to their *ex utero* development and transparency during embryogenesis, zebrafish can provide unique insights into developmental pathology. In contrast, mice are often raised into adulthood before culling, in order to allow for more comprehensive assessment of relevant phenotypes.

It is possible, therefore, that the mouse models discussed develop fetal phenotypes just as the zebrafish do, but these are compensated for at later stages and therefore missed during postnatal screening. Indeed, in human patients, AGS is often characterized by a period of pronounced symptomatic deterioration followed by stabilization and—in rare cases—small improvements (7, 69). However, patients rarely make a complete functional recovery, with the neuropathology and white matter lesions which first presented during early development observable throughout life. Likewise, longitudinal characterization of the *rnaset2* mutant zebrafish revealed white matter lesions and behavioral abnormalities which persisted into adulthood (37, 38). It is, therefore, unlikely that any fetal neuropathology in mice would fully rectify throughout development such that adults appeared neurologically normal at screening. Nonetheless, the discrepancy between mouse and zebrafish phenotypes highlights an important consideration when modeling disorders with such a prominent neurodevelopmental component.

### Methodological Considerations When Generating Animal Models

When assessing the validity of any animal model in recapitulating clinical phenotypes, it is important to consider the relevance of the model organism to patient genetics. Like many other interferonopathies, AGS and RNaseT2-deficient leukoencephalopathy are monogenetic disorders—as such, each of the animal models previously discussed disrupt the function or expression of a single gene linked to the human condition.

However, across mice and zebrafish, a range of genetic strategies have been utilized to generate disease models. It is notable that the mouse models discussed here have employed knockout approaches to mimic the loss-of-function mutations seen in many patients (excluding *IFIH1*)—resulting in animals with little-to-no expression of the relevant gene. As discussed elsewhere, these models have little relevance to the human genotype—with most patient mutations resulting in reduced expression of functioning or malfunctioning protein. Crucially, such extreme genotypes may limit the translational impact of these models in the development of therapies—particularly those which aim to reintroduce target proteins, such as enzyme-replacement or gene therapy. Against a constitutive knockout background, the reintroduced protein may initiate an immune response after being recognized as foreign—as has been reported in preclinical models of a closely related leukodystrophy, Alexander’s disease (70). In contrast, patients with some endogenous expression of these genes are perhaps less likely to develop an immune response to reintroduced proteins—making it difficult to predict the efficacy of such treatments based on these preclinical mouse models (8). Unlike their murine counterparts, many of the genetic tools used to generate zebrafish models of interferonopathies—such as antisense morpholino oligonucleotides and CRISPR/Cas9 gene editing—result in genotypes frequently more relevant to the human condition, by knocking down gene expression or generating mutated protein (rather than a constitutive knockout) (23, 38).

One might expect animals with a complete lack of relevant gene expression to present with an arguably more severe phenotype than those retaining some level of protein (whether this be reduced levels of functioning protein or dysfunctional enzyme). Indeed, this seems to be the case when considering *ADAR* mouse models—with animals with point mutations in the ADAR gene surviving slightly longer than complete knockouts (25–30). However, the same seems not to apply to animal models of *SAMHD1* and *RNaseT2* dysfunction. For each of these genes, constitutive knockout rodents fail to fully recapitulate the immune phenotypes reported in human conditions—with overtly normal development and survival (20–22, 36). In contrast, *samhd1* and *rnaset2* defective zebrafish develop robust neurological phenotypes relevant to the human condition—with *samhd1* models developing cerebral hemorrhage, while *rnaset2* mutants acquire white matter abnormalities and locomotor dysfunction (23, 37, 38). For each of these models, the genetic strategies utilized preserve some level of protein expression and, yet, their phenotypes are more severe—and arguably more relevant to the human condition—than their murine counterparts.

However, it should be noted that several studies in zebrafish have reported poor correlation between the phenotypes of mutants (i.e. those generated using CRISPR/Cas9) and morphants (those generated by morpholino)—with morphants often presenting with more severe phenotypes than mutants, even in the absence of any observable off-target effects (71, 72). In addition, subsequent research has suggested that the use of morpholinos themselves may induce an interferon-like response, with upregulation of ISGs reported across multiple published morphants (73). As such, it is possible that intrinsic limitations of morpholino-induced knockdown may account for the more severe phenotypes observed in *samhd1* zebrafish models relative to their murine counterparts (20–23). However, these findings still cannot account for the phenotypic differences between *RNaseT2* knockout rats and *rnaset2* mutant zebrafish—the latter of which has been validated using both ENU mutagenesis and CRISPR/Cas9 gene editing and bred to produce stable lines with comparable phenotypes (37, 38). As such, differences in methodology cannot entirely account for the differences in neurophenotypes reported in rodent and zebrafish models of type I interferonopathies.

In addition to the genetic modifications utilized to generate *in vivo* models, it should also be noted that there are substantial differences in the genetic backgrounds of zebrafish and mice used in experimental settings. For example, laboratory mice are highly inbred to reduce variability—particularly when characterizing phenotypes associated with single gene knockout—resulting in a single line which does not reflect the substantial genetic variability seen in human populations. In contrast, zebrafish are relatively outbred, leading to an accumulation of polymorphisms that vary from one animal to the next and perhaps more closely mimic the complex genetic make-up of humans than mice. The combined effect of these mutations may well act as a phenotypic modifier—resulting in intraspecies variability in pathology, as is seen in human AGS patients with mutations in the same gene (18). However, any increased variation in zebrafish models relative to mice still cannot account for the general trend towards greater neurological involvement in the fish compared to rodents. Thus, perhaps factors beyond genetics also serve to manipulate phenotypes in interferonopathy modeling.

### Mind the Microbiome—The Role of the Experimental Environment

When developing animal models of genetic disorders, often little attention is paid to the impact of the laboratory environment. Compared to their wild counterparts, lab mice and zebrafish live in a controlled environment in an effort to simplify our understanding of the relationship between genotype and phenotype. However, there are notable differences in the husbandry of zebrafish and mice—leading to arguably very distinct environment and pathogen exposure.

While zebrafish facilities around the world undoubtedly take great care in optimizing water quality in their aquaria, there is some evidence that pathogens are present in water across a large number of centers (74). A recent study reported evidence of a novel picornavirus-like pathogen transmitted *via* the environment—leading to spontaneous activation of interferon responses in otherwise healthy animals. Infection was associated with no overt phenotype, but rather was identified using an *isg15* transgenic reporter line. Intriguingly, evidence of widespread viral infection was identified in RNAseq datasets from 92 facilities across the world (74). It would therefore seem that “asymptomatic” zebrafish infection may be relatively commonplace in zebrafish research and act as somewhat of a confounding—although not necessarily unhelpful—factor in studying immune responses in these animals.

In contrast, mice live in a relatively “clean” environment compared to their zebrafish counterparts—with sterilization of bedding, food, and water being commonplace in murine husbandry. It is unsurprising, therefore, that lab mice are exposed to significantly fewer pathogens—including viruses—compared with their wild counterparts, contributing to notable differences in immune composition and antiviral response (75). This is in stark contrast to zebrafish and, of course, humans—for whom exposure to low virulence pathogens is commonplace throughout life and may even begin *in utero* (76–79), Perhaps, then, it is the sterile environment of laboratory mice—in which pathogen exposure is extremely low—which might explain immune phenotypes that are notably removed from the human condition.

Possible viral exposure is particularly relevant when modeling interferonopathies—a collection of disorders that have for so long been thought to mimic congenital viral infection and associated with genes involved in the human antiviral response. While active viral infection is usually excluded before a diagnosis of AGS or RNaseT2-deficient leukodystrophy can be made, it is possible that exposure to commonplace, low-virulence viruses could serve as a risk factor—or even a trigger—for activation of type I interferon response in patients that are already genetically predisposed to interferonopathies. It has been suggested that such viruses may be broadly linked to neurological pathologies in a manner that is complex and temporarily removed—this, too, may be the case for interferonopathies (80). Such viral infections may resolve without the development of overt phenotypes at the time of infection—instead, triggering the autoimmune response and resulting in downstream disability.

Perhaps, this previously unappreciated role of viral infection as a trigger for interferonopathy can explain why mice, in general, develop somewhat milder phenotypes, while zebrafish—with virus exposure even during larval stages—go on to develop similar pathology to that which we see in humans.

While viral exposure may be particularly relevant to the interferonopathies—with type I IFNs primarily considered for their role in antiviral response—bacterial infection is also known to trigger type I interferon response (81). In mice, deletion of IFNAR (the type I IFN receptor) has been shown to both protect against and exacerbate infection with different bacterial species—demonstrating a clear, albeit complex, role of bacteria in triggering type I IFN (82, 83). Likewise, in zebrafish, colonization of germ-free larvae with bacteria has also been shown to upregulate the interferon-stimulated genes, among other innate immunity-associated transcripts (84). It is conceivable, therefore, that environmental exposure to bacteria could also act as a trigger for interferonopathy pathology in zebrafish and humans in a similar manner to viruses—further exacerbating differences between murine and zebrafish pathology.

There have been numerous calls for mice to be raised in pathogen-rich environments in order to increase the impact of immune research following a crisis in translation that extends beyond inferonopathy modeling (66–68, 85). Indeed, research has suggested that exposing lab mice to a greater number of environmental pathogens may result in immune responses that better mimic human phenotypes (68). So-called wildling mice—mice born to wild mothers but with the same genetic background as conventional laboratory animals—better predicted patient response to immune-related therapies in clinical trials compared to conventional lab animals (68). While the precise viral exposure of these wildling mice was not assessed, these animals were maternally exposed to a more diverse microbial population than lab mice—suggesting life-long exposure to pathogens increases the face validity of mouse models in recapitulating human disease.

One might therefore expect that raising interferonopathy mouse models with greater pathogen exposure—or inducing viral infection—in early life may result in a more relevant neuroinflammatory phenotype. It should be noted that preliminary experiments inducing immune challenge in both *RNaseH2* mutant and *SAMHD1* knockout mice failed to find any difference in response compared to wildtype animals. *SAMHD1^-/-^* mice produced normal levels of IFNα and IFN response following encephalomyocarditis viral infection, while *RNaseH2* mutant mice developed a similar clinical phenotype as their wildtype counterparts following induction of experimental autoimmune encephalomyelitis (16, 21). However, it should be noted that both of these immune challenges were induced in adult—rather than developing—animals, and that long-term downstream effects were not observed. In humans, congenital infection by HIV-1 is characterized by upregulation of interferon α alongside intracranial calcification and white matter abnormalities—a phenotype remarkably similar to that of AGS—suggesting the timing of infection may well modulate the severity of pathology (4, 86–88). Thus, it is still entirely possible similar immune challenges could trigger a downstream inflammatory phenotype in these mouse models if performed during embryonic development or in early postnatal stages.

If asymptomatic, low-virulence viral infection does in fact trigger interferonopathy in humans, this too may provoke the type I interferon response observed in zebrafish models. Indeed, upregulated transcription of *isg15—*an interferon-stimulated gene known to be involved in the zebrafish immune response and the very transgenic reporter line used to identify the novel picornavirus-like pathogen endemic to zebrafish facilities across the world—has been reported in *samhd1*, *adar* and *rnaset2* defective zebrafish models throughout development (23, 38, 74). After hatching (around 2 days post fertilization), zebrafish larvae may be particularly susceptible to viral infection of the brain due to lack of a functional blood brain barrier (BBB) (89). As in mammals, the zebrafish BBB is thought to develop and become functional in a spatiotemporal manner, with the hindbrain BBB becoming functional around four days post fertilization and the midbrain a day later (89). As such, it is feasible that viruses—or, at least, mediators of the antiviral response—are able to enter the larval brain and trigger interferon response. This mechanism could also trigger IFN in human patients—however, our understanding of human BBB formation is less well characterized. Although embryonic BBB is thought to develop and become functional *in utero*, there is some suggestion that full maturation (including inclusion of mature cell types in the neurovascular unit) does not occur until after birth and, even after maturation, pro-inflammatory cytokines are able to cross the BBB with possible deleterious effects (90, 91). Thus, even if the human brain is protected from direct viral infection, it is conceivable that patients with mutations in AGS-associated genes are already susceptible to activation of the interferon response such that the antiviral response initiated by systemic infection may be sufficient trigger neuropathology by infiltration of cytokines into the developing brain.

The role of virus exposure in the zebrafish interferon response could be further dissected by exploiting the *ex utero* development of zebrafish embryos to raise animals in a sterile environment. Bleaching zebrafish eggs at 24 h post-fertilization has been shown to prevent productive viral infection throughout embryogenesis and is a strategy commonly used to raise embryos in a pathogen-free environment (74, 92). If bleached zebrafish mutants were to show an improved inflammatory phenotype compared to their conventionally reared counterparts, this would suggest a role for viral infection in triggering type I interferon response. Thus, careful modulation of the zebrafish microenvironment could be informative about the role of viral infection in triggering type I interferon response in autoimmune interferonopathy.

Recent publications in AGS have suggested that the autoimmune response observed in these patients is triggered by retroelement-derived nucleotides (7). If this is the case, manipulating viral exposure in animal models may well not alter their phenotypes at all. However, we believe that the reliably more severe neurological phenotypes present in the zebrafish compared to the mouse—despite similar genotypes and arguably greater evolutionary similarity between mice and humans than the zebrafish—suggest a prominent role for the environment in modulating pathogenesis of these disorders. These two schools of thought are by no means mutually exclusive: it is possible that viral infection and the presence of foreign nucleotides may provide the first trigger for a breakdown in self-tolerance, whereby individuals develop downstream autoimmune response to endogenous retroelements-derived nucleic acids which further drives pathology. Patients with AGS typically present with severe deterioration during the initial encephalopathic phase, but then stabilize and—in some cases—even show some small improvements (7, 69). Similarly, it has also been reported that some patients with *RNaseH2* and *RNaseT2* mutations may show normalization of interferon response over time: initially showing a positive interferon signature that later becomes negative at follow-up (18). If viral infection is a trigger for pathology, the initial flurry of antiviral response could explain this rapid deterioration and upregulation of ISGs, followed by subsequent stabilization as autoimmunity resolves. In contrast, if the trigger for pathology is truly endogenous in cause, one might expect a continued autoimmune response with consistent deterioration beyond the first year of life. Nonetheless, the points highlighted above suggest an additional layer of complexity in the pathogenesis of interferonopathies—or at least, their animal models—beyond simply genetics.

## Summary and Future Perspectives

Type I interferonopathies are a group of severe, life-limiting disorders—characterized by a disturbance of the homeostatic control of the interferon response and a range of downstream inflammatory phenotypes. With such profound effect on development and survival, interferonopathies with neurological involvement—including AGS and RNaseT2-deficient leukoencephalopathy—are particularly debilitating. Yet, despite their devastating effects, much remains to be understood about these disorders and, crucially, how to treat them.

Our understanding of these conditions and the development of novel therapies has thus far been limited by a lack of valid animal models (8). In this review, we have demonstrated consistent limitations in animal models across both species in mimicking the human disease state in AGS. However, mouse models in particular are limited in their recapitulation of the human neurological phenotype.

While there are several key differences between these species specifically relating to each of the AGS-associated genes, we propose that the disparity between rodent and fish models reflects the differing laboratory environments in which these animals are raised, and the corresponding effects on the immune system. Laboratory mice live in relatively sterile environments, and as such have an immune system largely removed from their wildtype counterparts. In contrast, both zebrafish and humans are exposed to a number of pathogens—including viruses—throughout early development: we believe this exposure is essential in modulating the development of interferonopathy neuropathology.

We propose that an initial viral stimulus may serve as the trigger for type I interferon response in AGS and RNaseT2-deficient leukoencephalopathy in human patients and corresponding zebrafish models, leading to subsequent autoimmune pathology due to a compromised genetic background. The absence of viral triggers in lab mice could explain why these animals do not develop the neuroinflammation central to AGS pathology, while the zebrafish—exposed to viruses throughout embryogenesis—develop somewhat more robust neurological pathology. Subsequent work may further explore the effects of viral stimuli in AGS models across both species.

Nonetheless, the vastly different phenotypes between zebrafish and rodent models with mutations of the same gene highlight the importance of model choice, methodological considerations and, perhaps most importantly, pathogen exposure when modeling disorders of the immune system. Future research must carefully consider how these unseen pathogens—or lack thereof—influence pathology if we are to ever understand the complex gene-environment interactions that form human immune response in interferonopathies and beyond.

## Author Contributions

HR performed the literature search, wrote and revised the manuscript, and designed the graphical figures. PK advised and critically revised the manuscript. NH conceived the study, critically revised and finally approved the manuscript. All authors contributed to the article and approved the submitted version.

## Funding

HR is supported by a studentship from the MRC Discovery Medicine North (DiMeN) Doctoral Training Partnership (MR/N013840/1). PK is supported by a New Investigator Grant from the MRC (MR/T03291X/1) and the Stroke Association (TSA LECT 2017/02).

## Conflict of Interest

The authors declare that the research was conducted in the absence of any commercial or financial relationships that could be construed as a potential conflict of interest.

## Abbreviations

AGS, Aicardi Goutières syndrome; CRISPR, clustered regularly interspaced short palindromic repeats; DNA, deoxyribonucleic acid; HIV, human immunodeficiency virus 1; IFN, interferon; ISG, interferon stimulated gene; LINE-1, long interspersed nuclear element 1; MRI, magnetic resonance imaging; RNA, ribonucleic acid; RTI, reverse transcriptase inhibitor; SLE, systemic lupus erythematosus.
